# Vitamin D Boosts Alendronate Tail Effect on Bone Mineral Density in Postmenopausal Women with Osteoporosis

**DOI:** 10.3390/nu13061878

**Published:** 2021-05-31

**Authors:** Antonino Catalano, Federica Bellone, Domenico Santoro, Peter Schwarz, Agostino Gaudio, Giorgio Basile, Maria Carmela Sottile, Sabrina Atena Stoian, Francesco Corica, Nunziata Morabito

**Affiliations:** 1 Unit and School of Geriatrics, Department of Clinical and Experimental Medicine, University of Messina, Policlinico “G. Martino”, Via C. Valeria, 98125 Messina, Italy; fbellone@unime.it (F.B.); basileg@unime.it (G.B.); mariac83sottile@gmail.com (M.C.S.); atenastoian@gmail.com (S.A.S.); coricaf@unime.it (F.C.); nmorabito@unime.it (N.M.); 2 Department of Endocrinology and Diabetes and Bone-Metabolic Research Unit, Rigshospitalet, University of Copenhagen, Blegdamsvej 9, 2100 Copenhagen, Denmark; peter.schwarz@regionh.dk; 3 Unit and School of Nephrology, Department of Clinical and Experimental Medicine, University of Messina, Policlinico “G. Martino”, Via C. Valeria, 98125 Messina, Italy; domenico.santoro@unime.it; 4 Unit and School of Internal Medicine, Department of Clinical and Experimental Medicine, University of Catania, Policlinico “G. Rodolico”, Via S. Sofia 78, 95123 Catania, Italy; agostino.gaudio@gmail.com

**Keywords:** bisphosphonates, alendronate, vitamin D, osteoporosis, postmenopausal, drug holiday

## Abstract

Vitamin D modulates bisphosphonate (BP) efficacy, but its contribution to bone mineral density (BMD) after BP discontinuation is not known. To address this topic, we performed a retrospective analysis of postmenopausal women exposed to alendronate (ALN) to treat osteoporosis who regularly continued the supplementation of cholecalciferol or calcifediol at recommended doses. In the ninety-six recruited women (age 61.1 ± 6.9 years), ALN was administered for 31.2 ± 20.6 months and then discontinued for 33.3 ± 18.9 months. The modification of 25(OH)D serum levels over time was associated with a change of alkaline phosphatase (r = −0.22, *p* = 0.018) and C-terminal collagen type 1 telopeptide (r = −0.3, *p* = 0.06). Women in the tertile of the highest increase in 25(OH)D level showed a 5.7% BMD gain at lumbar spine, that was twice as great in comparison with participants with a lower 25(OH)D variation. At a multiple regression analysis, BMD change was associated with time since menopause (ß = 2.28, SE 0.44, *p* < 0.0001), FRAX score for major fracture (ß = −0.65, SE 0.29, *p* = 0.03), drug holiday duration (ß = −2.17, SE 0.27, *p* < 0.0001) and change of 25(OH)D levels (ß = 0.15, SE 0.03, *p* = 0.0007). After ALN discontinuation, improving the vitamin D status boosts the ALN tail effect on BMD.

## 1. Introduction

Bisphosphonates (BPs) are the most common anti-resorptive drugs used for the treatment of osteoporosis [1]. In trials of 3–4 years durations, these drugs have shown to increase bone mineral density (BMD), maintain or improve mechanical bone strength and, ultimately, reduce the rate of fragility fractures [2,3,4,5,6,7,8]. The optimal long-term osteoporosis treatment is uncertain. Studies of the longer-term use of alendronate (ALN, up to 10 years), zoledronic acid (ZOL, up to 9 years) and risedronate (RIS, up to 7 years) have shown that in postmenopausal women who discontinue their use after 3–5 years, some benefits, including a reduced bone loss and a reduction in vertebral fractures, are retained [9,10,11,12]. Recent increased concerns regarding a possible association of BPs with the risk of rare severe adverse events such as osteonecrosis of the jaw (ONJ) and atypical femur fractures (AFF), have suggested that one should try to limit the duration of BP treatment, especially among post-menopausal women at low risk, i.e., no former osteoporotic fracture and BMD increase to a T-score > −2.5 SD [13,14]. Additionally, BPs bind to hydroxyapatite in bone and can remain there for many years, ensuing a residual pharmacological activity after their discontinuation [14,15]. ALN and ZOL are characterized by a more persistent effect after discontinuing treatment and this would allow for a more prolonged antifracture efficacy [15]. Thus, drug holidays may be recommended for patients previously exposed to BPs, in particular for those considered not to be at high risk for fracture [14]. Vitamin D mainly contributes to calcium homeostasis and preserves musculoskeletal health [16,17,18,19,20,21]. Vitamin D deficiency, defined as serum 25-hydroxyvitamin D (25(OH)D) < 50 nmol/L or 20 ng/mL, is quite common and occurs in <20% of the population in northern Europe, in 30–60% in western, southern and eastern Europe and up to 80% in Middle East countries. Severe deficiency (serum 25(OH)D < 30 nmol/L or 12 ng/mL) is found in >10% of Europeans [22]. Several cross-sectional studies and randomized controlled trials supported a role of vitamin D supplementation in terms of the reduction in falls as well as of hip and other fractures among people at risk for vitamin D deficiency [16,17,18]. BPs were originally introduced to treat osteoporosis based on randomized, placebo-controlled trials in which a vitamin D repletion was mandatory and vitamin D supplements were administered as an additional treatment [2,3,4,5,6,7,8]. Moreover, in postmenopausal women with osteoporosis, it was reported that adequate vitamin D repletion appears necessary to maximize the response to osteoporotic treatment in terms of both BMD changes and anti-fracture efficacy [23,24,25,26,27]. Nevertheless, in a real practice scenario, adherence to vitamin D supplementation has been observed to constantly decrease over the duration of osteoporotic treatment, possibly impairing the efficacy of treatment per se in terms of reduction in fractures [24,28]. Optimal vitamin D repletion has been advocated before initiation and during the BP treatment period, but insufficient data exist about the role of vitamin D status on BMD after BP discontinuation. Since ALN is the widest used BP to treat osteoporosis [1] and because no evidence is available on the role of vitamin D status after ALN discontinuation, the aim of our study was to evaluate in osteoporotic postmenopausal women whether vitamin D status may play a role on the tail effect of ALN—i.e., after ALN discontinuation—on preserving BMD, which represents a surrogate of bone strength [29].

## 2. Materials and Methods

This is a retrospective study retrieving postmenopausal women who received a prescription of ALN for osteoporosis between the years 2006 and 2016. Patients were included if they maintained treatment with ALN for at least 6 months and for ≥80% of the total time at doses and dosing intervals approved for the management of post-menopausal osteoporosis. Further inclusion criteria were the availability of BMD measurements before ALN treatment and after ALN discontinuation. Women were excluded if they had previously been treated with anti-resorptive or anabolic bone agents for osteoporosis (e.g., other BPs, denosumab, strontium ranelate, selective estrogen receptor modulators or teriparatide), or were systemic glucocorticoid users or were suffering from medical disorders associated with bone loss, including renal insufficiency (creatinine clearance < 30 mL/min), active malignancy, hyperthyroidism, hyperparathyroidism and malabsorption syndromes. All participants received ALN treatment at a daily dose of 70 mg (tablet or liquid) once a week. The recommended daily allowance of calcium was reached with calcium carbonate supplementation (500–600 mg, daily) in women with an estimated poor dietary calcium intake. Oral cholecalciferol (25,000 UI every 2 weeks) or calcifediol (100 to 125 µg weekly) was used as vitamin D supplementation regularly all over the study period. BMD was measured through a dual-energy X-ray absorptiometry (DXA) densitometer (Hologic Discovery Wi) at the lumbar spine (L1–L4) in AP projection and at the femoral neck. In accordance with the manufacture’s instructions, the DXA densitometer was calibrated daily, and its coefficient of variation (CV) was 0.5% using the standard phantom. Markers of bone turnover, such as alkaline phosphatase (ALP) and C-terminal collagen type 1 telopeptide (CTX) were assessed. Serum (s-) 25(OH)D, s-calcium, s-phosphorus, s-creatinine were also measured. S-25(OH)D was determined by high performance liquid chromatography; s-CTX was assessed using the Elecsys 2010 Immunoassay System (Roche, Basel, Switzerland) with intraassay CVs of 1.6% to 3% and interassay CVs of 1.3% to 4.3%. S-calcium, s-phosphorus, s-ALP and s-creatinine were measured through routine procedures. The study was carried out in compliance with the ethical standards of our institutional research committee “Comitato Etico Messina” and with the 1964 Declaration of Helsinki and its later amendments. All the participants signed an informed consent before they entered the study. MedCalc software (version 10.2.0.0; MedCalc Software, Mariakerke, Belgium) was used to perform statistical analyses. The Kolmogorov–Smirnov test allowed one to assess the normal distribution of the data. Values were reported as mean ± SD or median (interquartile range). The unpaired t test or the Mann–Whitney test were applied for inter-group comparisons, whereas the paired t test or the Wilcoxon matched rank sum test for paired data were determined for within-group comparisons as appropriate. The association between the studied variables was tested through the Spearman correlation coefficient (rho), while the relationship between a dependent variable and one or more explanatory variables was examined by multiple regression analysis. A *p* value < 0.05 reflected a statistical significance for all the tests used. All reported *p* values were two-sided.

## 3. Results

Out of 1686 patients who had received a prescription of ALN in the period from 2006 to 2016, 96 women (age 61.1 ± 6.9 years) were included in the present investigation. Reasons for treatment discontinuation included the uncomfortable way of drug administration (30%), lack of motivation (25%), side effects (20%) the excessive suppression of bone turn-over (20%), or others (5%). The main clinical features of these participants are shown in Table 1.

**Figure 1:**
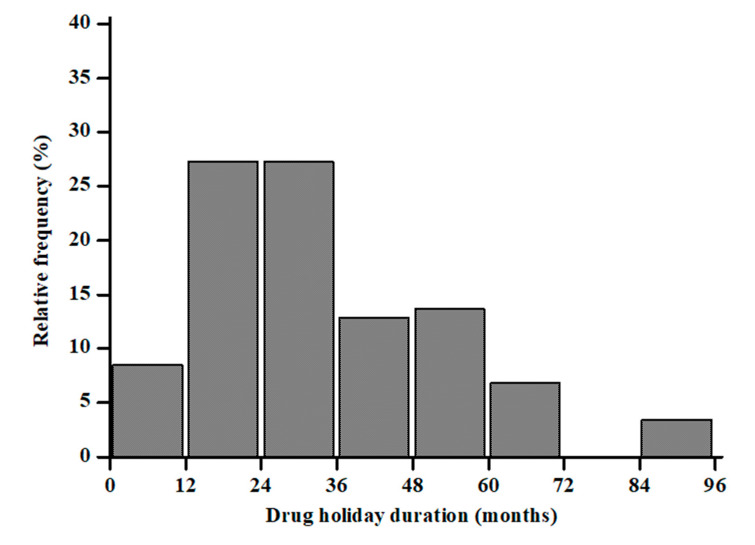
Distribution of months under treatment with ALN in a cohort of postmenopausal women.

The 10-year probability of major and hip fractures was 18.3 ± 11.5 and 8.6 ± 10.5%, respectively. Sixty-two (65%) showed morphometric vertebral fractures. Overall, ALN was previously administered for 31.2 ± 20.6 months and then discontinued for 33.3 ± 18.9 months. Figure 1 shows the relative frequency of the participants’ ALN treatment.

**Figure 2:**
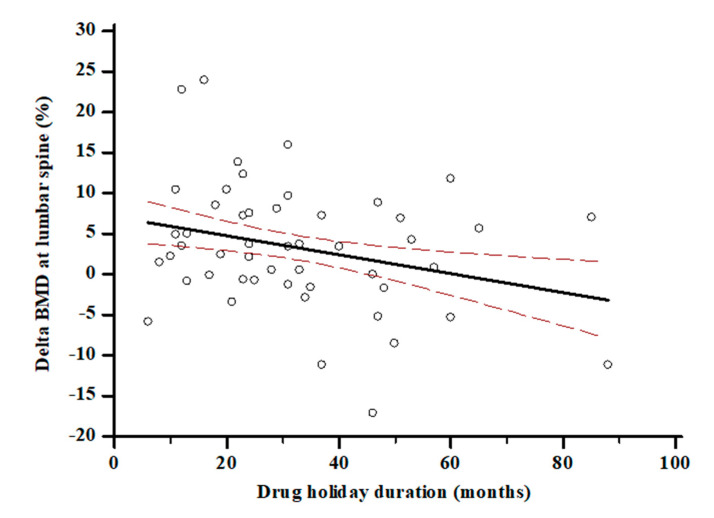
Association between ALN discontinuation—i.e., drug holiday—and BMD change at lumbar spine. (r = −0.27; *p* = 0.005).

**Figure 3:**
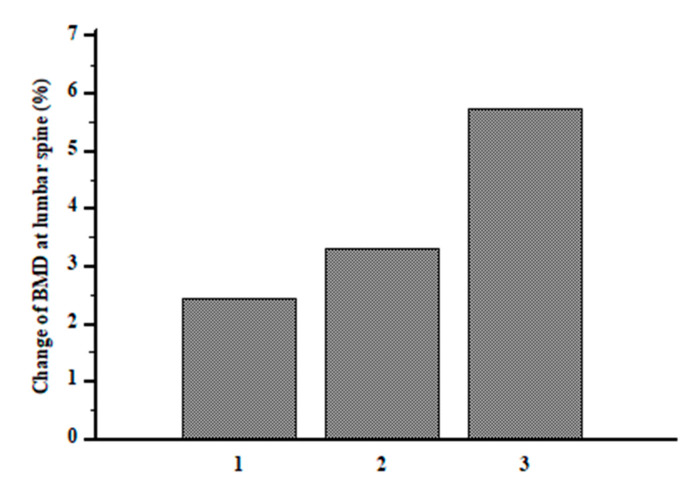
Change of BMD at lumbar spine after ALN discontinuation according to tertiles of serum 25(OH)D variation.

The length of discontinuation was inversely associated with time since menopause (r = −0.32; *p* = 0.0005) and with loss of BMD at lumbar spine (r = −0.27; *p* = 0.005) (Figure 2). BMD at the lumbar spine (0.70 ± 0.32 vs. 0.86 ± 0.11 g/cm^2^, *p* < 0.0001) and femoral neck (0.53 ± 0.28 vs. 0.63 ± 0.19 g/cm^2^, *p* < 0.001) increased significantly over the observation period in the entire cohort. BMD at lumbar spine increased significantly within each tertile (*p*^all^ < 0.05); on the other hand, BMD at femoral neck increased only in ΔD3 tertile (0.45 ± 0.31 vs. 0.63 ± 0.16 g/cm^2^, *p* = 0.002) and remained not significantly changed in tertile ΔD1 and ΔD2 tertile (*p*^all^ > 0.05). S-25(OH)D at baseline was not associated with the BMD change neither at lumbar spine (r = 0.03, *p* = 0.08) nor femoral neck (r = 0.001, *p* = 0.7) after ALN discontinuation. The modification of s−25(OH)D levels over time was inversely associated with the change of ALP (r = −0.22, *p* = 0.018) and s-CTX levels (r = −0.3, *p* = 0.06). Moreover, the participants in the ΔD3, the one with the largest increase in s-25(OH)D levels, showed a 5.7% BMD gain, that was twice as great in comparison with participants with the lower 25(OH)D variation in ΔD1 (Figure 3). At a multiple regression analysis, after correcting for ALN treatment duration, bone turnover marker modifications, BMI and age at menopause, the BMD change at lumbar spine was significantly associated with time since menopause (ß = 2.28, SE 0.44, *p* < 0.0001), FRAX score for major fracture (ß = −0.65, SE 0.29, *p* = 0.03), ALN discontinuation duration (ß = −2.17, SE 0.27, *p* < 0.0001) and change of s-25(OH)D levels (ß = 0.15, SE 0.03, *p* = 0.0007).

## 4. Discussion

In a cohort of 96 postmenopausal osteoporotic women who had been treated with ALN, we found an association between the tail effect of ALN on BMD with the modification of s-25(OH)D levels. Particularly, after ALN discontinuation, changes in BMD at the lumbar spine were positively influenced by the favorable modification of vitamin D status over the observation period, and the contribution of vitamin D also remained significant after adjustment for potential confounding variables. S-25(OH)D levels reflect the total body vitamin D status from all sources including dietary intake and sun exposure with endogenous synthesis [30,31]. Although there is no consensus on optimal serum levels of 25(OH)D, most experts consider a 25(OH)D level less than 50 nmol/L (20 ng/mL) to be indicative of vitamin D deficiency in the general population [22]. Values above the cut-off of 75 nmol/L (30 ng/mL) are considered advisable for patients under BPs [26]. However, more than half of postmenopausal women taking medication for osteoporosis were observed to show s-25(OH)D levels below 75 nmol/L (30 ng/mL) [23]. Adami et al. found that over the course of treatment with ALN, RIS or raloxifene, the rate of incident fracture was significantly lower among patients who took also calcium and vitamin D supplements, in comparison with patients who did not take any of the above-mentioned supplements [24]. In a related study, the odds of incident fractures were significantly higher in vitamin D deficient women as compared to those who were vitamin D replete [25]. Furthermore, Barone et al. highlighted that lumbar spine BMD increased more in patients treated with ALN plus calcitriol, the active form of vitamin D, compared to ALN alone [32]. An adequate vitamin D supplementation has even been proven to blunt the acute phase response after BP administration [33]. In our study, all the participants were supplemented with vitamin D (cholecalciferol or calcifediol) in order to prevent vitamin D deficiency. S-25(OH)D levels, as measured at baseline, were not associated with BMD change both at lumbar spine and femoral neck after ALN discontinuation. This finding may reflect at least in part the change of vitamin D status over time in women taking vitamin D supplementation, although compliance with vitamin D is not the sole reason for variation in circulating s-25(OH)D levels. Variations of s-25(OH)D levels, in fact, may depend on intestinal absorption, liver 25-hydroxylation of cholecalciferol and tissue metabolism to inactive metabolites, which are processes influenced by medical treatments or dietary and physiologic correlates [31]. An inadequate vitamin D status may affect bone metabolism through osteoclastic bone resorption and bone loss induced by enhanced PTH secretion [32,34,35]; it may also negatively impact muscle mass and function and consequently increase the risk of falls, contributing to fragility fractures [21]. In our study, participants were free from incident fractures and maintained or improved their BMD, a surrogate of bone strength, despite ALN discontinuation. The participants who best improved their vitamin D status, i.e., those in the ΔD3 tertile, obtained the most favorable BMD gain. This finding supports the need to maintain a supplementation of vitamin D along with and after ALN discontinuation in order to boost the residual ALN effect on bone tissue. BPs have been proven to improve BMD and reduce fracture incidence in short-term randomized controlled trials of up to 3 years [2,3,4,5,6,7,8]. Only a few studies have demonstrated the effectiveness of BPs beyond this time span so far [9,10,11,12]. Discontinuing BPs due to worries of rare adverse events, especially long-term side effects, has been proposed after a regular course of treatment of 3–5 years, but no recommendations as for maintaining the ALN tail effect have been advanced. In our study, we show vitamin D status may synergize with the ALN still tied to bone in order to also produce BMD gains after ALN discontinuation. Accordingly, we further observed a significant association between change of s-25(OH)D levels and changes of markers of bone turnover, which once again suggest that an improved vitamin D status may contribute to the suppression of bone turnover and to BMD preservation. The persistent suppression of bone remodelling allows a progressive mineralization of bone tissue, and this explains the slight increase in BMD after ALN discontinuation [15]. Conversely, a drug holiday approach is not recommended for other anti-resorptive medications with different mechanisms of action, and particularly with denosumab, whose discontinuation rapidly increases bone loss and raises the risk of vertebral fractures [36]. The discontinuation of other BPs such as risedronate and ibandronate is associated with a fast loss of the acquired BMD improvement, and with these two BPs, discontinuation should not exceed 6 months. For other BPs, in which no residual effects are expected, a drug holiday is usually not applied [36,37]. In these circumstances, the role of vitamin D status on the residual BPs effect remains unknown. Our research has limitations, including the retrospective design, the different duration of exposure to the ALN treatment and the different schedule of vitamin D supplementation. On one hand, these limits may make our findings challenging to interpret; on the other hand, due to the anti-fracture role of vitamin D, it seems unlikely, for ethical reasons, to design a longitudinal study to compare the tail effect of BPs, after their discontinuation, in vitamin D deplete (and not supplemented) vs. vitamin D replete (supplemented) patients with osteoporosis and high fracture risk. Nevertheless, these data reflect a real-life scenario and consequently provide further important knowledge.

## 5. Conclusions

Vitamin D is recognized to reduce the fracture risk in subjects at risk for hypovitaminosis D and to improve the outcome of osteoporosis medications. ALN is a BP with a long half-life in bone, which continues its action for some time even after its discontinuation. For the first time, we provide evidence for the role of vitamin D in boosting the ALN tail effect on BMD in postmenopausal women treated for osteoporosis followed by treatment discontinuation.

## Figures and Tables

**Table 1:** Main clinical characteristics of postmenopausal women discontinuing alendronate treatment according with change of vitamin D levels.

	Whole Population (n = 96)	ΔD1 (n = 31)	ΔD2 (n = 33)	ΔD3 (n = 32)
Main clinical characteristics				
Age (years)	61.1 ± 6.9	58.9 ± 6.9	62.5 ± 6.6 *	62.1 ± 7.0
BMI (kg/m^2^)	25.7 ± 3.4	24.5 ± 2.5	26.6 ± 4.5	25.6 ± 2.9
Age at menopause (years)	47.4 ± 4.9	47.7 ± 5.1	47.3 ± 5.6	47.4 ± 4.3
Time since menopause (years)	13.2 ± 6.8	10.6 ± 5.9	14.4 ± 6.0 *	14.2 ± 7.9 ^#^
Ten year probability of major fractures (%)	18.3 ± 11.51	16.5 ± 8.9	20.9 ± 11.7	17.9 ± 13.7
Ten year probability of hip fractures (%)	8.6 ± 10.5	6.2 ± 4.9	10.9 ± 10.9 *	9.0 ± 13.8
ALN treatment duration (months)	31.2 ± 20.6	35.7 ± 23.5	31.3 ± 22.0	26.1 ± 14.4 ^#^
Drug holiday (months)	33.3 ± 18.9	34.1 ± 16.9	31.9 ± 20.7	34.4 ± 19.4
DXA measurements				
L1–L4 BMD (g/cm^2^)	0.70 ± 0.32	0.74 ± 0.28	0.69 ± 0.31	0.66 ± 0.37
Femoral neck BMD (g/cm^2^)	0.53 ± 0.28	0.63 ± 0.16	0.57 ± 0.26	0.45 ± 0.31^#^
Bone metabolism				
CTX (ng/mL)	0.57 ± 0.33	0.40 ± 0.28	0.58 ± 0.32	0.66 ± 0.38
ALP (U/L)	76.23 ± 35.39	68.18 ± 19.00	63.6 ± 19.3	99.76 ± 50.96 *^,#^
25(OH)D (ng/mL) at the time of ALN discontinuation	33.75 ± 15.47	46.64 ± 17.2	33.62 ± 7.18 *	21.84 ± 8.97 *^,#^
25(OH)D (ng/mL) at the end of the observation period (†)	44.81 ± 13.89	38.58 ± 10.79	43.53 ± 8.68 *	51.12 ± 11.62 *^,#^

Data are expressed as mean ± SD. Tertiles derived from a post hoc analysis evaluating percent change of 25(OH)D: ΔD1 < ΔD2 < ΔD3. ALN = alendronate; DXA = dual-energy X-ray absorptiometry; BMD = bone mineral density; CTX = C-terminal collagen type 1 telopeptide; ALP = alkaline phosphatase; 25(OH)D = 25-hydroxyvitamin D; † = observation period after ALN discontinuation was 33.3 ± 18.9 months; * = *p* < 0.05 vs. ΔD1; # = *p* < 0.5 vs. ΔD2.

## Data Availability

The data presented in this study are available on request from the corresponding author.
